# Fabrication of Carbonate Apatite Block through a Dissolution–Precipitation Reaction Using Calcium Hydrogen Phosphate Dihydrate Block as a Precursor

**DOI:** 10.3390/ma10040374

**Published:** 2017-03-31

**Authors:** Kanji Tsuru, Ayami Yoshimoto, Masayuki Kanazawa, Yuki Sugiura, Yasuharu Nakashima, Kunio Ishikawa

**Affiliations:** 1 Department of Biomaterials, Faculty of Dental Science, Kyushu University, 3-1-1 Maidashi, Higashi-ku, Fukuoka 812-8582, Japan; yoshimoto.ayami.638@m.kyushu-u.ac.jp (A.Y.); ysugiura@dent.kyushu-u.ac.jp (Y.S.); ishikawa@dent.kyushu-u.ac.jp (K.I.); 2 Department of Orthopaedic Surgery, Graduate School of Medical Sciences, Kyushu University, 3-1-1 Maidashi, Higashi-ku, Fukuoka 812-8582, Japan; masacana@ortho.med.kyushu-u.ac.jp (M.K.); yasunaka@ortho.med.kyushu-u.ac.jp (Y.N.)

**Keywords:** bone replacement, carbonate apatite, calcium hydrogen phosphate dihydrate, dissolution–precipitation reaction

## Abstract

Carbonate apatite (CO_3_Ap) block, which is a bone replacement used to repair defects, was fabricated through a dissolution–precipitation reaction using a calcium hydrogen phosphate dihydrate (DCPD) block as a precursor. When the DCPD block was immersed in NaHCO_3_ or Na_2_CO_3_ solution at 80 °C, DCPD converted to CO_3_Ap within 3 days. β-Tricalcium phosphate was formed as an intermediate phase, and it was completely converted to CO_3_Ap within 2 weeks when the DCPD block was immersed in Na_2_CO_3_ solution. Although the crystal structures of the DCPD and CO_3_Ap blocks were different, the macroscopic structure was maintained during the compositional transformation through the dissolution–precipitation reaction. CO_3_Ap block fabricated in NaHCO_3_ or Na_2_CO_3_ solution contained 12.9 and 15.8 wt % carbonate, respectively. The diametral tensile strength of the CO_3_Ap block was 2 MPa, and the porosity was approximately 57% regardless of the carbonate solution. DCPD is a useful precursor for the fabrication of CO_3_Ap block.

## 1. Introduction

Bone apatite is different from hydroxyapatite (HAp; Ca_10_(PO_4_)_6_(OH)_2_), but it is similar to carbonate apatite (CO_3_Ap), which contains 6–9 wt % carbonate in the apatitic structure [1]. CO_3_Ap is denoted as A-type or B-type on the basis of the substitution site of carbonate ions in the apatitic lattice. Type A CO_3_Ap (CO_3_-for-OH substitution) can be prepared at high temperature (1000 °C) whereas B-type CO_3_Ap (CO_3_-for-PO_4_ substitution) can be prepared by precipitation or by hydrolysis at low temperature [1]. B-type CO_3_Ap is a candidate for artificial bone substitute since its structure and crystallinity are similar to natural bone. However, B-type CO_3_Ap decomposes thermally at sintering temperatures. As a result, sintering methods cannot be used for the fabrication of CO_3_Ap bone substitute. In the era from the 1970s to 1980s, sintered HAp, which is free of carbonate, showed excellent tissue response and good osteoconductivity [2,3,4]. Therefore, sintered HAp has been widely used as an artificial bone substitute [5,6,7]. One major drawback of sintered HAp is its stability, and grafted HAp is hardly replaced by new bone.

CO_3_Ap block has been fabricated using dissolution–precipitation and precursors such as calcite [CaCO_3_] [8,9], gypsum [CaSO_4_·2H_2_O] [10,11], and α-tricalcium phosphate (TCP; [α-Ca_3_(PO_4_)_2_]) [12,13,14]. When osteoclasts were incubated on the surface of CO_3_Ap blocks, osteoclastic resorption pits similar to bone were observed [8]. Because of the absence and presence of osteoclastic resorption, sintered HAp is not replaced by new bone, whereas CO_3_Ap block is. CO_3_Ap can upregulate differentiation of osteoblasts more than HAp [15]. Moreover, CO_3_Ap has demonstrated higher osteoconductivity than sintered HAp.

Although CO_3_Ap block is a promising artificial bone replacement, the studies conducted so far to identify the ideal precursor have been limited. There are requirements for the precursor involved in the fabrication of CO_3_Ap block. First, a precursor should be a block. Compositional transformation from the precursor to CO_3_Ap block occurs through a dissolution–precipitation reaction, maintaining the macroscopic structure of the precursor. Second, the precursor should have at least one component of CO_3_Ap such as calcium, phosphate or carbonate. Third, the precursor should be in a metastable phase and have suitable solubility in the solution used for the dissolution–precipitation reaction. If solubility is too low, it takes too long to fabricate the CO_3_Ap block from the precursor because of the rate of the dissolution–precipitation reaction. If the solubility is too high, the precursor cannot maintain its structure, and CO_3_Ap powder is formed instead of CO_3_Ap block. For example, calcium chloride cannot be a precursor because its solubility is too high even though it contains calcium.

Dicalcium phosphate dihydrate [DCPD; CaHPO_4_·2H_2_O] is a candidate for the fabrication of CO_3_Ap block. DCPD block can be fabricated from a setting reaction, i.e., DCPD-forming cement [16,17,18,19]. DCPD contains both calcium and phosphate in its composition. DCPD block may have an unsuitable solubility for the compositional transformation to CO_3_Ap through a dissolution–precipitation reaction because the solubility of DCPD is higher than that of other precursors such as calcite, α-TCP, and gypsum. Besides, DCPD is an acidic calcium phosphate. For the compositional transformation to CO_3_Ap through a dissolution–precipitation reaction using a precursor block, the solution around the precursor block should be supersaturated with respect to CO_3_Ap. An acidic environment is unsuitable for the liquid to be supersaturated with respect to CO_3_Ap.

In this study, the feasibility of the fabrication of CO_3_Ap block by compositional transformation through dissolution–precipitation reaction using DCPD block as a precursor was evaluated. DCPD block was prepared using a setting reaction of β-TCP and monocalcium phosphate monohydrate [MCPM: Ca(HPO_4_)_2_·H_2_O].

## 2. Results

**Figure 1:**
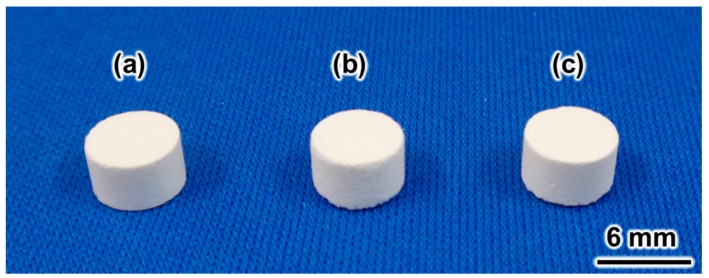
Photographs of set samples. (**a**) Set sample made with the setting reaction of the β-tricalcium phosphate and monocalcium phosphate monohydrate mixture, and samples obtained by immersion in (**b**) 2 M NaHCO_3_ and (**c**) 2 M Na_2_CO_3_ solutions at 80 °C for 14 days.

Figure 1 summarizes the photographs of the set samples. Figure 1a shows the set sample resulting from the setting reaction of the β-TCP and MCPM mixture. Figure 1b is the sample obtained by immersion of the set sample in 2 M NaHCO_3_ at 80 °C for 14 days. Figure 1c is the sample obtained by immersion of the set sample in 2 M Na_2_CO_3_ at 80 °C for 14 days. Samples maintained their macroscopic structure after the immersion in carbonate solutions regardless of the carbonate solution.

**Figure 2:**
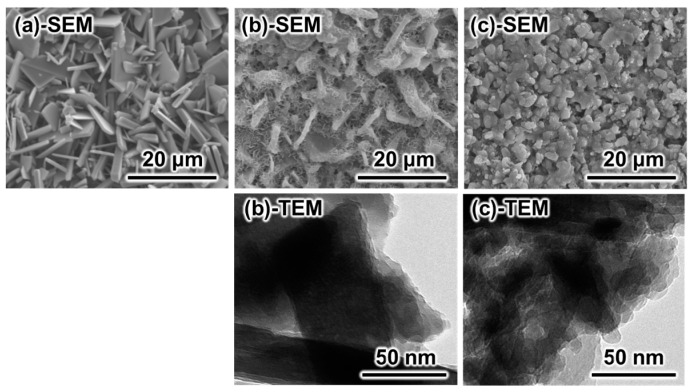
Representative SEM and TEM images of samples. (**a**) Set sample made with the setting reaction of the β-tricalcium phosphate and monocalcium phosphate monohydrate mixture and set samples immersed in (**b**) 2 M NaHCO_3_ and (**c**) 2 M Na_2_CO_3_ solutions at 80 °C for 14 days.

Figure 2 summarizes representative scanning electron microscope (SEM) and transmission electron microscope (TEM) images of a set sample made with the setting reaction of the β-TCP and MCPM mixture, after the sample was immersed in 2 M NaHCO_3_ at 80 °C for 14 days, and 2 M Na_2_CO_3_ at 80 °C for 14 days. Although the samples maintained their macroscopic structure as shown in Figure 1, the morphology of the crystals was different before and after immersion in the carbonate solution. The sample made with the setting reaction of the β-TCP and MCPM mixture showed plate-like crystals, which are similar to the typical morphology of DCPD crystals (Figure 2a). When the sample was immersed in 2 M NaHCO_3_ at 80 °C for 14 days, crystals with two different morphologies were present in the sample (Figure 2b-SEM). One crystal showed a plate-like structure similar to DCPD crystals. On the surface of the plate-like crystals, small needle-like crystals were present. However, when immersed in 2 M Na_2_CO_3_ at 80 °C for 14 days, polygon-like crystals were formed (Figure 2c-SEM). The TEM micrographs supported the results of the SEM observation.

**Figure 3:**
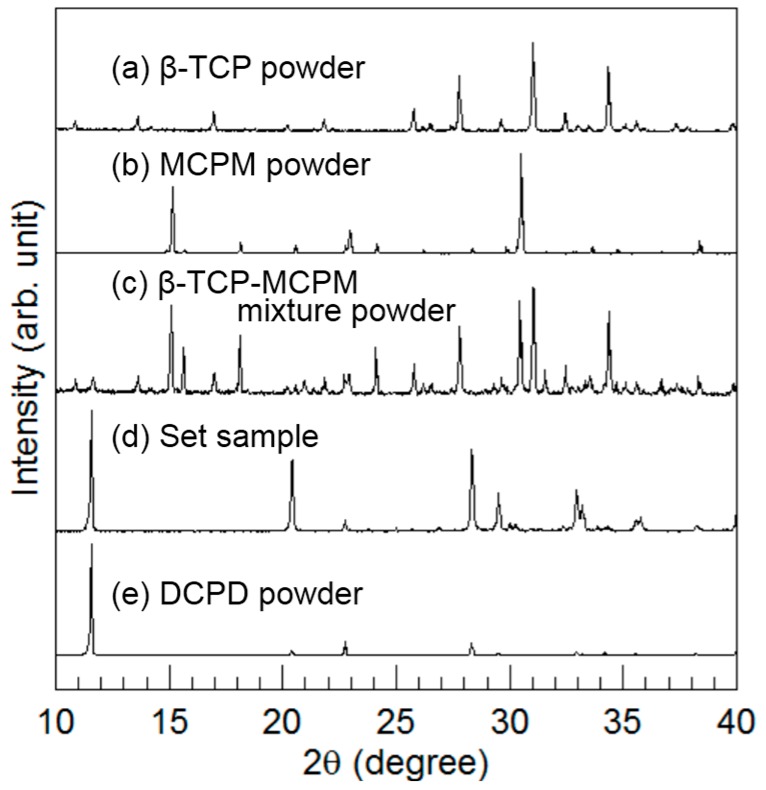
X-ray diffraction patterns of (**a**) β-tricalcium phosphate (TCP) powder; (**b**) monocalcium phosphate monohydrate (MCPM) powder; (**c**) β-TCP and MCPM mixed powder; (**d**) set sample made from the mixture of β-TCP and MCPM; and (**e**) calcium hydrogen phosphate dihydrate powder was used as a reference.

Figure 3 summarizes the powder XRD patterns. In addition to the XRD patterns of β-TCP powder, MCPM powder, β-TCP-MCPM mixture powder, and the set sample made from the mixture of β-TCP and MCPM, a standard DCPD pattern is shown to facilitate comparison. The mixture of β-TCP and MCPM became DCPD when exposed to water during the setting reaction.

Hereafter, the set sample made with the mixture of β-TCP and MCPM is referred to as the DCPD block.

**Figure 4:**
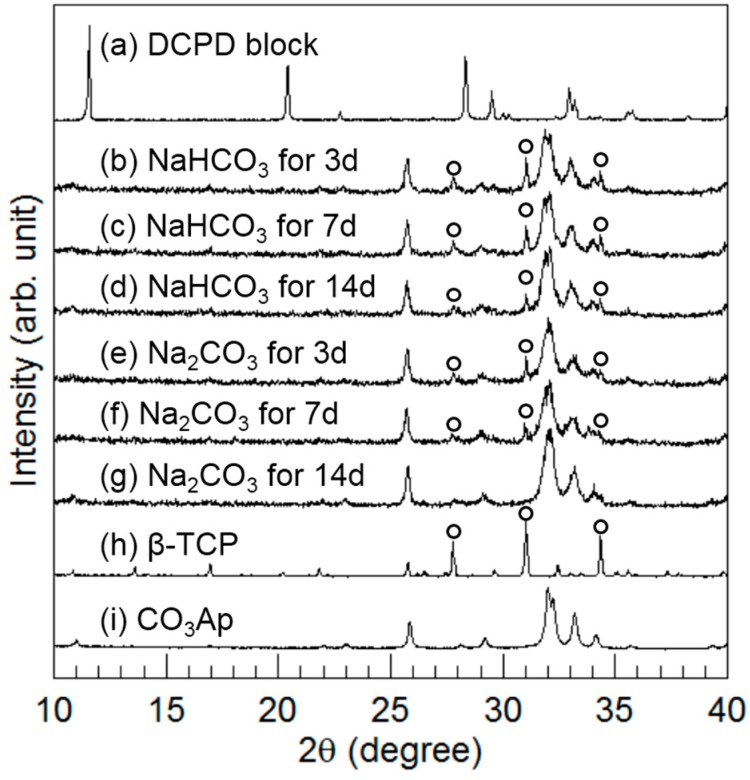
X-ray diffraction patterns of calcium hydrogen phosphate dihydrate block (**a**) before and after immersion in (**b**–**d**) 2 M NaHCO_3_ and (**e**–**g**) 2 M Na_2_CO_3_ solutions at 80 °C for up to 14 days. XRD patterns of (**h**) β-tricalcium phosphate and (**i**) CO_3_Ap are listed as references.

Figure 4 summarizes the XRD patterns of the DCPD block before and after immersion in 2 M NaHCO_3_ or 2 M Na_2_CO_3_ at 80 °C for up to 14 days. After 3 days of immersion, peaks similar to CO_3_Ap were detected, and the peaks assigned to DCPD disappeared. Furthermore, the peaks assigned to β-TCP (indicated by the open circles in Figure 4) were also detected regardless of sodium carbonate solution. The peak height of β-TCP corresponding to the amount of β-TCP decreased with immersion time. In the case of immersion in the NaHCO_3_ solution, the β-TCP peaks remained even after 14 days. However, in the case of immersion in the Na_2_CO_3_ solution, the peak height of β-TCP, which was lower than that for NaHCO_3_ immersion, decreased with time and disappeared within 14 days. Therefore, conversion to CO_3_Ap was faster in the case of Na_2_CO_3_ immersion than in the case of NaHCO_3_ immersion. In addition, the CO_3_Ap peaks became broad so that a low-crystalline CO_3_Ap was obtained in this method.

**Figure 5:**
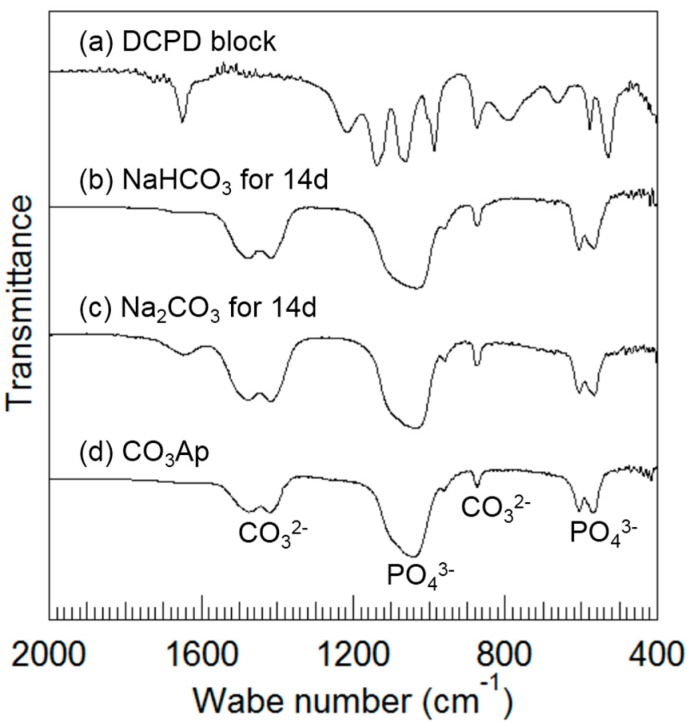
Fourier transform infrared spectra of calcium hydrogen phosphate dehydrate block (**a**) before and after immersion in (**b**) 2 M NaHCO_3_ and (**c**) 2 M Na_2_CO_3_ solutions at 80 °C for 14 days; (**d**) Spectrum of CO_3_Ap is used as a reference.

Figure 5 summarizes the FTIR spectra of the DCPD block (a) before and after immersion in (b) 2 M NaHCO_3_ at 80 °C for up to 14 days, and after immersion in (c) 2 M Na_2_CO_3_ at 80 °C for up to 14 days. The FTIR spectrum of (d) CO_3_Ap is shown for comparison. The frequencies at 567, 606, 1042, and 1092 cm^−1^ are assigned to PO_4_^3−^ groups [20], the frequencies at 875, 1418, and 1474 cm^−1^ are assigned to CO_3_^2−^ groups [21], and the frequencies at 640 cm^−1^ are assigned to OH^−^ groups [20]. FTIR spectra obtained after immersed in sodium carbonate solutions (Figure 5b,c) were similar to those of CO_3_Ap (Figure 5d) and different from those of DCPD (Figure 5a). A larger band due to low crystallinity was observed when the DCPD block was immersed in the carbonate solutions. The band at 1413 cm^−1^ observed in the obtained CO_3_Ap is reported to be assigned to B-type CO_3_Ap [1]. No peaks corresponding to OH^−^ groups were present. As a result, the obtained CO_3_Ap is expected to be AB-type CO_3_Ap.

Table 1 summarizes the carbonate content of DCPD blocks after immersion in 2 M NaHCO_3_ and 2 M Na_2_CO_3_ at 80 °C for up to 14 days. Both samples contained CO_3_. The carbonate contents of the DCPD block immersed in 2 M NaHCO_3_ and Na_2_CO_3_ at 80 °C for 14 days were 12.9% ± 0.5% and 15.8% ± 0.9%, respectively.

**Figure 6:**
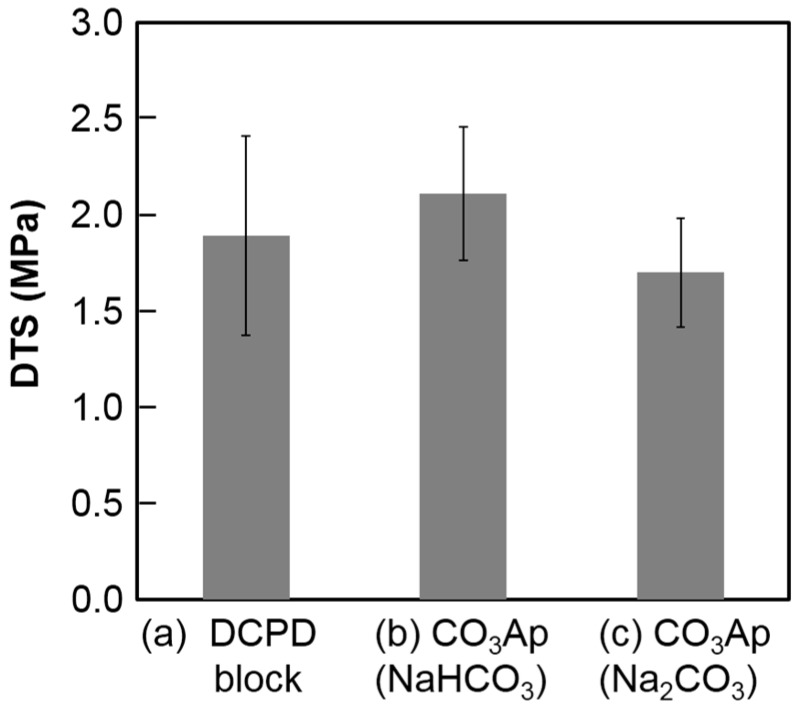
Diametral tensile strengths the calcium hydrogen phosphate dihydrate block (**a**) before and after immersion in (**b**) 2 M NaHCO_3_ and (**c**) 2 M Na_2_CO_3_ solutions at 80 °C for 14 days.

Figure 6 summarizes the DTS values of the DCPD block before and after immersion in the carbonate solutions. No statically significant difference was observed before and after immersion regardless of the carbonate solution.

**Figure 7:**
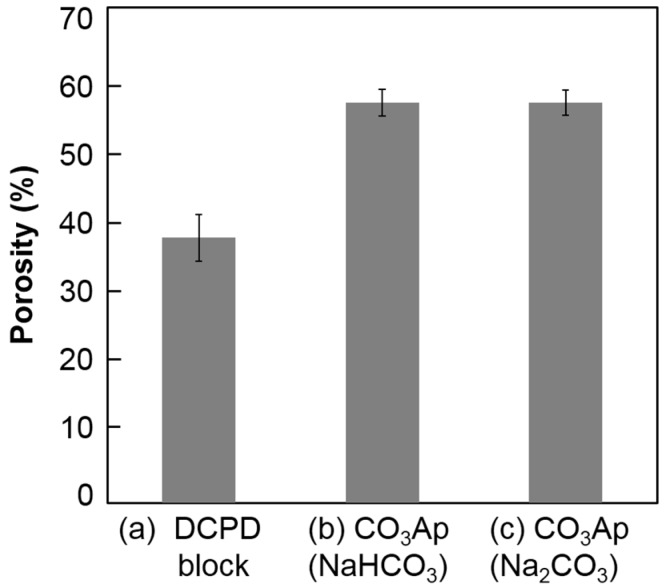
Porosity of the calcium hydrogen phosphate dihydrate block (**a**) before and after immersion in (**b**) 2 M NaHCO_3_ and (**c**) 2 M Na_2_CO_3_ solutions at 80 °C for 14 days.

Figure 7 summarizes the porosity of the DCPD block before and after immersion in the carbonate solutions. The porosity of the DCPD block was 37.4% ± 3.3%. After immersion in the carbonate solution, porosity increased to 56.5% ± 1.9% (NaHCO_3_ solution) and 56.6%± 1.8% (Na_2_CO_3_ solution). No statically significant difference was observed between the carbonate solutions.

## 3. Discussion

The results obtained in this study demonstrate that CO_3_Ap block can be fabricated though a dissolution–precipitation reaction using DCPD block as a precursor. The DCPD block satisfied the requirements for fabricating the CO_3_Ap block.

An ideal precursor is a block because the macroscopic structure is maintained during the dissolution–precipitation reaction. As shown in Figure 1a, the DCPD block can be made by a setting reaction of a mixture of β-TCP and MCPM or a setting reaction of DCPD-forming cement. The SEM observation shown in Figure 2a reveals that precipitated DCPD crystals interlock with each other during setting. The setting reaction of the DCPD block is also a dissolution–precipitation reaction. This microporous structure may be an ideal precursor for CO_3_Ap fabrication through a dissolution–precipitation reaction because the dissolution of the microporous block is faster than that of a dense block, and there is space for the formation of CO_3_Ap crystals.

The setting time of the DCPD-forming cement was very short in the absence of a retarder. Although the setting time can be regulated by adding retarders such as citric acid, pyrophosphate, or sulfuric acid, no retarder was introduced to the mixture of β-TCP and MCPM in the present study to simplify the precursor. 

DCPD contains both calcium and phosphate; thus, only carbonate is required for the fabrication of the CO_3_Ap block. Therefore, the DCPD block was immersed in NaHCO_3_ or Na_2_CO_3_ solution in the present study. The solution condition has a close relationship with the solubility. Although, the solubilities of DCPD in the NaHCO_3_ and Na_2_CO_3_ solutions are higher than other precursors such as calcite, calcium sulfate dihydrate, or α-TCP, the DCPD block was suitable for CO_3_Ap block fabrication because it was not dissolved during the reaction, and the macroscopic structure was retained as shown in Figure 1b,c. Because of the high solubility, the DCPD phase disappears as early as 1 day of immersion regardless of the carbonate solution. Faster compositional transformation from DCPD to CO_3_Ap is reasonable because this reaction proceeds through a dissolution–precipitation mechanism. β-TCP was present 1 day after immersion in the carbonate solutions as an intermediate phase. The amount of β-TCP was higher when the DCPD block was immersed in NaHCO_3_ solution than in Na_2_CO_3_ solution. The amount of β-TCP decreased with immersion time and disappeared completely when the DCPD block was immersed in Na_2_CO_3_ solution for 2 weeks. However, β-TCP remained, even after 2 weeks when immersed in NaHCO_3_ solution. This difference may be because of the pH of the solution. In other words, β-TCP is more stable at neutral pH. The appearance of β-TCP as an intermediate phase demonstrates the possibility to use β-TCP block as a precursor for CO_3_Ap block fabrication even though its solubility is much lower than that of α-TCP. Moreover, the β-TCP block may be fabricated in the solution. The β-TCP block fabricated in the solution may have a higher osteoconductivity than β-TCP block fabricated using a sintering process.

Different pH values of the carbonate solution cause different crystal morphologies as shown in Figure 2b,c. Slower dissolution of DCPD in NaHCO_3_ solution than in Na_2_CO_3_ solution may be the reason for this difference. In other words, DCPD crystals, shown in Figure 2a, dissolve quickly, and the solution around DCPD is highly supersaturated with respect to CO_3_Ap, leading to the formation of more CO_3_Ap nuclei, increasing CO_3_Ap formation. When the DCPD block is immersed in NaHCO_3_ solution, DCPD dissolves slowly and results in a less supersaturated solution with respect to CO_3_Ap. Therefore, the precipitation of CO_3_Ap occurs only on the surface of DCPD crystals, and CO_3_Ap crystals maintain the crystal structure of DCPD.

Since the CO_3_Ap block was prepared through a dissolution-precipitation reaction at low temperature (80°C), the FTIR spectra of the obtained CO_3_Ap block had a broad band, indicating low crystalline CO_3_Ap that was also confirmed by XRD results. The large band made it difficult to quantitatively determine the types of CO_3_Ap. The band at 1413 cm^−1^ observed in the obtained CO_3_Ap is reported to be assigned to B-type CO_3_Ap [1]. Therefore it would be B-type CO_3_Ap. However, no peaks corresponding to OH^−^ groups were present in the spectra of the obtained CO_3_Ap. As a result, not only B-type but also A-type might co-exist in the obtained CO_3_Ap. Moreover, a broad band near 875 cm^−1^ assigned to the CO_3_^2−^ group consists of three components, such as A-type (878 cm^−^^1^), B-type (871 cm^−^^1^) and CO_3_^2^^−^ formed from apatitic lattice (866 cm^−^^1^) [22]. Based on the results, we also have to consider the formation of CO_3_^2^^−^ from apatitic lattice such as that adsorbed on the surface of CO_3_Ap crystals.

The mechanical strength of the CO_3_Ap block in terms of DTS was higher (*p* < 0.05) when the DCPD block was immersed in NaHCO_3_ solution due to the crystal structure of CO_3_Ap. Plate-like CO_3_Ap shows high interlocking among the crystals. Although there was a slight difference in mechanical strength, there were no differences in porosity. The porosity of the CO_3_Ap block was approximately 57% regardless of the carbonate solution, which was lower than that of the DCPD block. The Ca/P ratio of DCPD is lower than that of CO_3_Ap, which has a Ca/P ratio around 2. Thus, DCPD needs to release PO_4_ and gain CO_3_ to transform its composition to CO_3_Ap. A high porosity indicates that the amount of released PO_4_ is higher than the amount of incorporated CO_3_ from the solution. Therefore, CO_3_Ap fabricated by compositional transformation through a dissolution–precipitation reaction using a precursor maintains its macroscopic structure but cannot maintain its microscopic structure.

## 4. Materials and Methods

### 4.1. Preparation of the DCPD Block

The DCPD block was formed by the setting reaction of β-TCP and MCPM. β-TCP powder (Taihei, Osaka, Japan) and MCPM powder (Sigma–Aldrich Co., Saint Louis, MO, USA) were mixed with methanol (Wako Pure Chemical, Osaka, Japan) at a Ca/P molar ratio of 1.0. The methanol was allowed to evaporate at room temperature, and the mixture was placed into a split plastic steel mold 6 mm in diameter and 3 mm in height. Water was added dropwise until a water to powder weight ratio of 0.001 was reached. The samples were kept at 100% humidity for 24 h prior to testing.

### 4.2. Compositional Transformation from the Precursor Block to the CO_3_Ap Block

The obtained DCPD block was immersed in 2 M NaHCO_3_ or 2 M Na_2_CO_3_ solution at 80 °C for up to 14 days. In this treatment, ten DCPD blocks were immersed in each sodium carbonate solution of 500 mL. After the treatment, the DCPD blocks were removed from the sodium carbonate solution and rinsed with distilled water.

### 4.3. Powder X-ray Diffractometry

The crystal phase of the obtained samples was detected by powder X-ray diffraction (XRD) analysis. The specimen was ground into a fine powder and used for the analysis. XRD patterns were recorded with an X-ray diffractometer (D8 Advance, Bruker AXS GmbH., Karlsruhe, Germany) using monochromatized X-ray (CuKα: λ = 0.1542 nm) operating at the condition of 40 kV and 40 mA. The diffraction angle was continuously scanned from 10° to 60° in 2θ at a scanning rate of 2°/min. A range of 10°–40° is shown in the figures because no relevant peaks were observed in the excluded region.

### 4.4. Fourier Transform Infrared Spectroscopy

Fourier transform infrared (FTIR) spectroscopy was performed with an FTIR spectrometer (FT/IR-6200, JASCO, Tokyo, Japan) using the KBr method over a wavenumber range of 400–2000 cm^−1^. A spectral resolution of 4 cm^−1^ was employed to examine structural changes.

### 4.5. Electron Microscopy

The surface morphology of the obtained samples was observed by a scanning electron microscope (SEM; S-3400N, Hitachi High-Technologies Co., Tokyo, Japan) at 15 kV of accelerating voltage after gold–palladium coating by a magnetron sputtering machine (MSP-1S, Vacuum Device Co., Ibaraki, Japan). The fine structure of the obtained samples was observed by a transmission electron microscope (TEM; JEM-1400Plus, JEOL Co., Tokyo, Japan) at 100 kV of accelerating voltage.

### 4.6. Porosity Measurement

The porosity the obtained specimen was calculated using the bulk density of the sample (*d*_sample_) and the theoretical density of HAp (*d*_HAp_ 3.16 g/cm^3^) [23], as shown in Equation (1).

Porosity (%) = *d*_HAp_ − *d*_sample_/*d*_HAp_ × 100 (1)

### 4.7. Carbonate Contents

Carbonate contents were estimated from the wt % of carbon in the CO_3_Ap block. A CHN coder (MT-6; Yanako Analytical Instruments, Kyoto, Japan) was used to analyze the wt % of carbon.

### 4.8. Mechanical Strength Measurement

The mechanical strength of disk-shaped samples was evaluated in terms of their diametral tensile strength (DTS). After drying the samples at 60 °C for 24 h, their diameter and thickness were measured using a micrometer (MDC-25MU, Mitutoyo Co. Ltd., Kawasaki, Japan), and the samples were weighed using a microbalance. The samples were crushed with a universal testing machine (AGS-J, Shimadzu, Kyoto, Japan) at a constant crosshead speed of 10 mm/min. The mean DTS value for eight samples was calculated and expressed as mean ± standard deviation.

### 4.9. Statistical Analysis

For statistical analysis, one-way analysis of variance and Fisher’s LSD method, as a post-hoc test, were performed using Kaleida Graph 4. We consider that *p* < 0.05 is statistically significant.

## 5. Conclusions

CO_3_Ap block was fabricated using DCPD block as a precursor by immersing the block in carbonate solutions. β-TCP forms as an intermediate phase during the transformation of the DCPD block to the CO_3_Ap block by compositional transformation through a dissolution–precipitation reaction. Further studies are awaited based on the results obtained in this initial study.

## Figures and Tables

**Table 1:** Carbonate content of DCPD blocks after immersion in 2 M NaHCO_3_ and Na_2_CO_3_ at 80 °C for 14 days.

Solution	CO_2_ Contents (wt %)
NaHCO_3_	12.9 ± 0.5
Na_2_CO_3_	15.8 ± 0.9
